# An internal ribosome entry site in the 5′ untranslated region of epidermal growth factor receptor allows hypoxic expression

**DOI:** 10.1038/oncsis.2014.43

**Published:** 2015-01-26

**Authors:** T E Webb, A Hughes, D S Smalley, K A Spriggs

**Affiliations:** 1 School of Pharmacy, University of Nottingham, Nottingham, UK

## Abstract

The expression of epidermal growth factor receptor (EGFR/ERBB1/HER1) is implicated in the progress of numerous cancers, a feature that has been exploited in the development of EGFR antibodies and EGFR tyrosine kinase inhibitors as anti-cancer drugs. However, EGFR also has important normal cellular functions, leading to serious side effects when EGFR is inhibited. One damaging characteristic of many oncogenes is the ability to be expressed in the hypoxic conditions associated with the tumour interior. It has previously been demonstrated that expression of EGFR is maintained in hypoxic conditions via an unknown mechanism of translational control, despite global translation rates generally being attenuated under hypoxic conditions. In this report, we demonstrate that the human EGFR 5′ untranslated region (UTR) sequence can initiate the expression of a downstream open reading frame via an internal ribosome entry site (IRES). We show that this effect is not due to either cryptic promoter activity or splicing events. We have investigated the requirement of the EGFR IRES for eukaryotic initiation factor 4A (eIF4A), which is an RNA helicase responsible for processing RNA secondary structure as part of translation initiation. Treatment with hippuristanol (a potent inhibitor of eIF4A) caused a decrease in EGFR 5′ UTR-driven reporter activity and also a reduction in EGFR protein level. Importantly, we show that expression of a reporter gene under the control of the EGFR IRES is maintained under hypoxic conditions despite a fall in global translation rates.

## Introduction

Epidermal growth factor receptor (EGFR, also ErbB-1 or HER1) is an important drug target and prognostic indicator in many cancers. It is a transmembrane glycoprotein tyrosine kinase. Its primary function is to stimulate Akt-, MAPK- or JNK-mediated cellular proliferation in response to a range of ligands including transforming growth factor alpha and the family of EGFs (reviewed in the study by Oda *et al.*^1^).

Overexpression of EGFR has been strongly linked to poor prognosis in a large number of cancers including breast, head, neck, ovarian, cervical, bladder and oesophageal cancers (reviewed in the study by Nicholson *et al.*^2^). Mutations in EGFR have also been shown to be important, for example a subset of tumours possess a constitutively active truncated version of EGFR.^3^ Although there is a strong relationship between the frequency of this mutation and poor prognosis, possibly more importantly is overexpression of EGFR with nearly half of glioblastomas displaying significantly elevated levels of the wild-type receptor.^3, 4, 5, 6, 7, 8, 9^ A major mechanism by which EGFR is overexpressed is gene amplification, as demonstrated in colorectal, pulmonary, bile duct and soft tissue cancers.^10, 11, 12, 13^ In each of these instances however, amplification was not the only cause of the overexpression, with a marked discrepancy between gene dosage and EGFR protein levels.^14^ While a proportion of this overexpression may be attributable to mutations that cause the transcriptional upregulation of the EGFR gene,^15, 16, 17, 18^ it has also been shown that that EGFR protein levels are upregulated in response to both hypoxia and the activation of hypoxia-inducible factor 2α without observing either mutational events or changes in EGFR mRNA levels.^19^ Increased EGFR activity is also associated with Alzheimer’s disease, and inhibiting EGFR reverses amyloid beta-induced memory loss in mice.^20^ The untranslated regions (UTRs) of an mRNA have major roles in the translational control of its expression. While the 3′ UTR of EGFR has been investigated and shown to be a target for microRNA-induced suppression under certain conditions, the 5′ UTR remains largely unstudied.^21^

In general, cells respond to hypoxia by decreasing protein synthesis rates.^22, 23^ One mechanism used to accomplish this in the short term involves the phosphorylation of the translation initiation factor eIF2α. When hypo-phosphorylated, the function of eIF2α is to assist in the binding of the initiator transfer RNA to the 40S ribosomal subunit by forming a ternary complex with GTP. Phosphorylation of eIF2α inhibits this process, thereby acting as a brake on global translation rates.^24, 25^

Since internal ribosome entry site (IRES)-mediated translation initiation does not involve the binding of the 5′ cap, it is favoured under certain conditions, like hypoxia, that inhibit eukaryotic initiation factor 4E (eIF4E) function.^26, 27, 28^ Despite this reduced requirement for eIF4E, it has been demonstrated that the IRESs belonging to the human genes *c-myc*, N-*myc* and BiP have a strong requirement for eIF4A function for their expression.^29, 30, 31^ It has been suggested that this requirement indicates that the structure of these IRESs needs ‘remodelling’ by eIF4A before they are able to function, similar to the processing required by the encephalomyocarditis virus IRES.^30, 32, 33, 34^

We tested the EGFR 5′ UTR for IRES activity using a dicistronic reporter system and found that it was able to initiate the translation of the downstream cistron. Experiments on the EGFR 5′ UTR reporter using the eIF4A inhibitor hippuristanol found that the EGFR IRES has a high requirement for eIF4A. Finally, we show that the EGFR 5′ UTR allows hypoxic expression of a downstream cistron under conditions where global translation is compromised. These findings strongly support the idea that the induction of EGFR expression in response to hypoxic conditions is attributable to the presence of a previously unidentified IRES within its 5′ UTR.

## Results and Discussion

It has previously been shown that EGFR protein levels are maintained under hypoxic conditions despite a global decrease in protein synthesis rates, but the mechanism responsible is not known.^19^ Moreover, EGFR has recently been shown to be a regulator of hypoxic microRNA maturation through phosphorylation of AGO2.^35^

**Figure 1:**
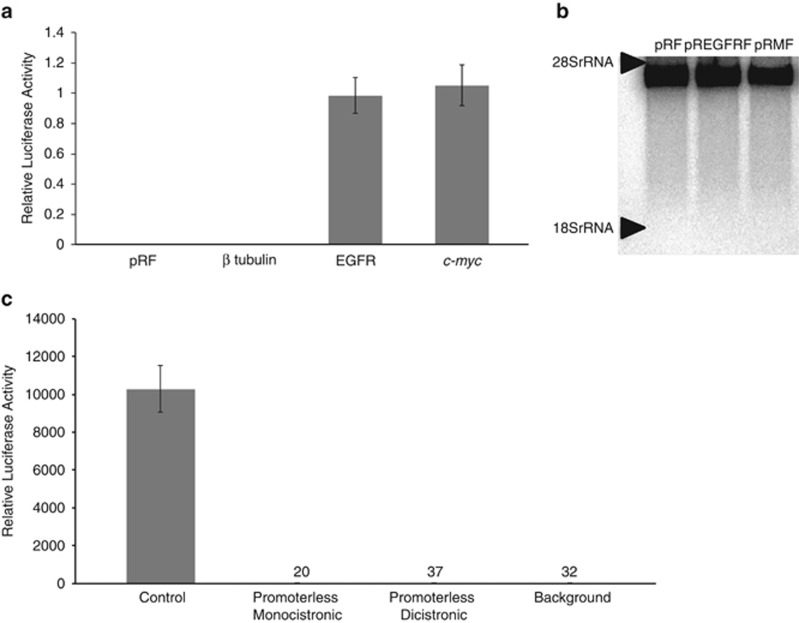
The EGFR 5′ UTR sequence permits the expression of the downstream open reading frame of a dicistronic reporter construct without exhibiting cryptic promoter or splicing activity. The UTRs of β tubulin and EGFR were synthesised by GenScript (Piscataway, NJ, USA) to match the nucleotide sequences with accession numbers NM_178014.2 and NM_201283.1, respectively. These sequences were amplified by PCR using primers containing SpeI restriction sites upstream and NcoI sites downstream, the UTRs were then cloned into pRF between these sites and the resulting plasmid was termed pR-EGFR-F. The dicistronic reporter containing the c-myc 5′ UTR (pRMF) was as described in a previous paper.^36^ The promoter-less version of the dicistronic reporter was created by cloning the two luciferase cistrons, flanking the EGFR 5′ UTR into a plasmid, which did not contain a promoter sequence. The promoter-less version of the monocistronic reporter was made by cloning the EGFR 5′ UTR into p15hp in place of the hairpin^39^ and then disabling the cytomegalovirus promoter by AseI digest. (**a**, **c**) Twenty-four-well plates were seeded with SH-SY5Y cells at a density of 50 000 cells/well. The following day, cells were transfected with 200 ng/well of the plasmids described above using FuGene 6 (Roche, Mannheim, Germany). The growth medium was changed after 6 h and the cells were cultured for the following 24 h. Luciferase expression within the cells was then quantified using a Dual Luciferase Assay Kit (Promega, Madison, WI, USA) following manufacturer’s instructions. Mean and s.d. of at least three replicates are shown. (**b**) A Northern blot was performed on lysates from HeLa cells previously transfected with pRF, pR-EGFR-F or pRMF using a probe complementary to the firefly luciferase open reading frame. The dicistronic EGFR reporter plasmid generates a single mRNA.

The EGFR 5′ UTR was cloned between the *Renilla* and firefly luciferase open reading frames of pRF and the resultant construct (pR-EGFR-F) was transfected into human neuroblastoma-derived SH-SY5Y cells. Parallel control transfections of pRF (a negative control lacking any IRES element^36, 37^), pRMF (containing the c-myc IRES^36^) and pR-Tub-F (containing the β tubulin 5′ UTR, which lacks IRES activity). The EGFR 5′ UTR permitted the expression of the downstream cistron in SH-SY5Y cells to a similar extent as the well-validated *c-myc* 5′ UTR, whereas the 5′ UTR of β tubulin did not (Figure 1a.). This effect was also observed in HeLa, Huh7 and MCF7 cells (data not shown). To determine whether cryptic splicing was occuring (which could lead to functional firefly luciferase transcripts in the absence of IRES activity) Northern analysis was performed using a radio-labelled probe against the firefly luciferase coding region. This confirmed the presence of a single luciferase containing transcript of the appropriate size in the transfected cells (Figure 1b). To confirm that the observed activity was not due to transcription of the firefly luciferase open reading frame driven by a cryptic promoter in the EGFR 5′ UTR sequence, the cytomegalovirus promoter was removed from the dicistronic constructs by restriction digestion, and the resultant promoter-less constructs were transfected into SH-SY5Y cells to confirm the absence of endogenous cryptic promoter activity in any of the 5′ UTR sequences (Figure 1c). We therefore conclude that the expression of the downstream cistron is directed by an IRES in the EGFR 5′ UTR.

**Figure 2:**
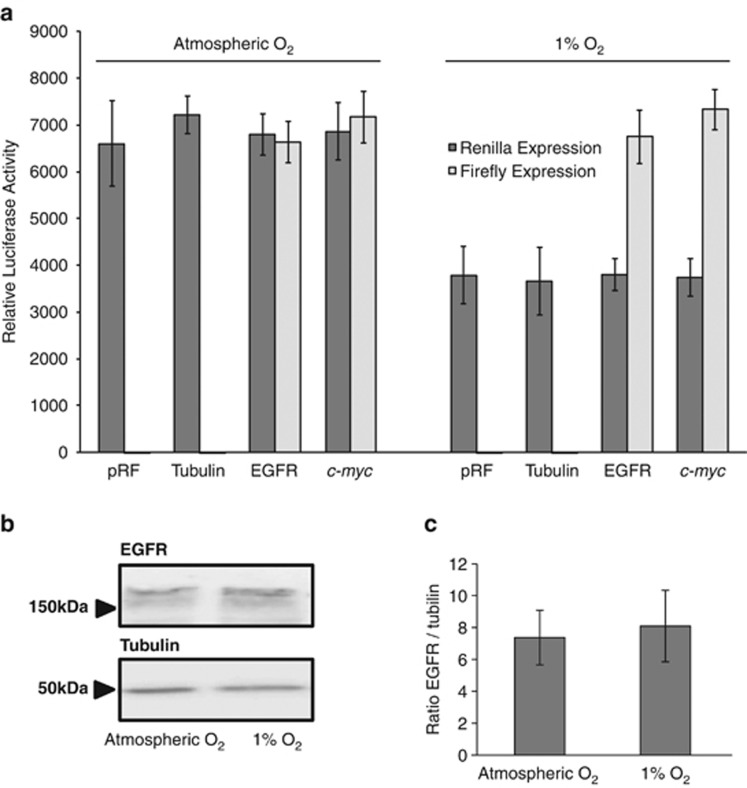
Expression of a reporter gene preceded by the 5′ UTR of EGFR is maintained under hypoxic conditions despite a fall in control reporter expression. (**a**) Twenty-four-well plates were seeded with SH-SY5Y cells at a density of 50 000 cells/well. The following day, cells were transfected with 200 ng/well of the plasmids described in the legend of Figure 1 using FuGene 6. The growth medium was changed after 6 h, and for the following 24 h one of the plates was kept in a control incubator set to atmospheric O_2_ levels. The other plate was incubated in a ProOx 110 (BioSpherix Ltd., Lacona, NY, USA) hypoxic chamber which O_2_ restricted to 1%. Luciferase expression within the cells was then quantified using a Dual Luciferase Assay Kit following manufacturer’s instructions. Mean and s.d. of at least three replicates are shown. (**b**) Six-well plates were seeded with SH-SY5Y cells at a density of 3 × 10^5^ cells/well. The following day, cells were incubated for 24 h at either atmospheric or 1% O_2_ levels. EGFR and tubulin protein levels were determined by western blotting using primary antibodies ab6012 (Abcam, Cambridge, UK) and sc-7396 (Santa Cruz, Dallas, TX, USA). (**c**) Four replicates of the blot described in Figure 2b were quantified using ImageJ (US National Institutes of Health, Bethesda, Maryland, USA). The data are presented as EGFR expression normalised to tubulin expression. No significant differences in EGFR expression were seen between normoxic (atmospheric) and hypoxic (1% O_2_) cells.

Having identified an IRES in the EGFR 5′ UTR, we wished to determine whether this could further explain previous observations that increases in EGFR protein can occur without parallel increases in mRNA.^19^ Hypoxic conditions (1% O_2_) caused a reduction in control *Renilla* luciferase expression to ~50% of its control value while the expression of the firefly open reading frames preceded by the EGFR and c-myc 5′ UTR sequences were maintained (Figure 2). The response of the *c-myc* IRES to hypoxia has been documented previously and it is included here as a positive control.^38^

**Figure 3:**
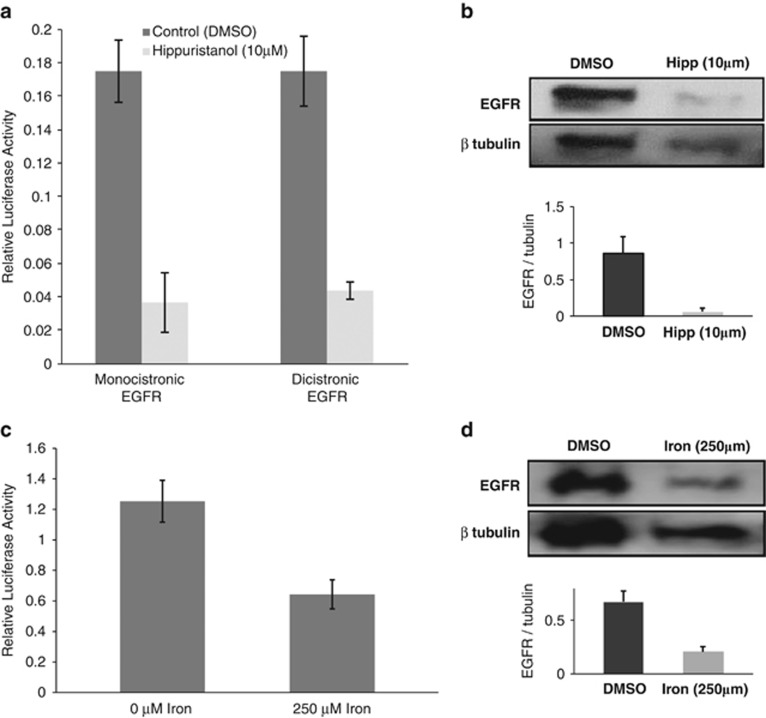
(**a**) EGFR expression is reduced by hippuristanol treatment and iron treatment via its 5′ UTR. Ten micromolar Hippuristanol (or DMSO) was added to HeLa cells, which were transfected with reporter plasmids 4 h previously. The creation of the reporter plasmid is described previously (Figure 1.). Luciferase expression was quantified 24 h later and firefly values were normalised to control *Renilla* values (pGL4.80cmv was co-transfected with the monocistronic reporter). Mean and s.d. of at least three replicates are shown. (**b**) Cellular lysate was also western blotted for EGFR and β tubulin using Abcam antibodies ab2430 and ab6046, respectively; representative blots are pictured. Quantification of three replicates of this experiment was performed using ImageJ and the mean expression levels of EGFR normalised to tubulin were calculated. (**c**, **d**) The experiment was repeated as above substituting hippuristanol with 250 μM of ammonium iron citrate.

To begin to characterise the requirements of the EGFR IRES, we added 10 μM hippuristanol (a potent and specific small molecule inhibitor of eIF4A^31^) to HeLa cells transfected with the monocistronic and dicistronic reporters. eIF4A is a DEAD-box helicase involved in unwinding secondary structure in 5′ UTRs and is required for efficient cap-independent translation of a number of transcripts.^31, 39^ This treatment with hippuristanol revealed a significant reduction in EGFR 5′ UTR-mediated reporter expression (*P*=0.007; Figure 3a). The dependency of the EGFR IRES on eIF4A function is also demonstrated by western blot, with hippuristanol causing a dramatic reduction in the protein level of EGFR, relative to a β tubulin loading control in HeLa (*P*=0.01; Figure 3b).

Iron has been implicated in the function of several IRESes including those in APP,^40^ HCV^41^ and cytoplasmic serine hydroxymethyltransferase.^42^ Iron stress is also known to induce the unfolded protein response,^37^ which leads to a global reduction in protein synthesis caused by eIF2alpha phosphorylation.^23^ EGFR protein levels specifically have also been shown to respond to iron, however, the mechanisms responsible have not previously been identified.^43, 44^ We therefore wished to test whether iron has a specific effect on EGFR IRES function beyond the general downregulation of cap-dependent translation caused by eIF2alpha phosphorylation. SH-SY5Y, HeLa and Huh7 cells were treated with 250 μM ammonium iron citrate or control, and a marked reduction in expression of firefly luciferase expression was observed from reporters driven by the EGFR 5′ UTR (Figure 3c) compared with controls. This inhibition was paralleled by a significant reduction in endogenous EGFR expression following treatment of HeLa cells with 250 μM ammonium iron citrate (*P*=0.02; Figures 3d and e) compared with expression of beta-tubulin. We have not been able to identify a recognisable iron response element in the EGFR 5′ UTR primary sequence, however, it is apparent that the EGFR 5′ UTR confers susceptibility to iron stress beyond that explained by the modest global decrease in translation or proliferation exemplified by beta-tubulin protein levels (Figure 3d). There are precedents for iron levels influencing IRES activity^40, 42^ and further work is required to confirm whether similar mechanisms are at work in EGFR translational control.

**Figure 4:**
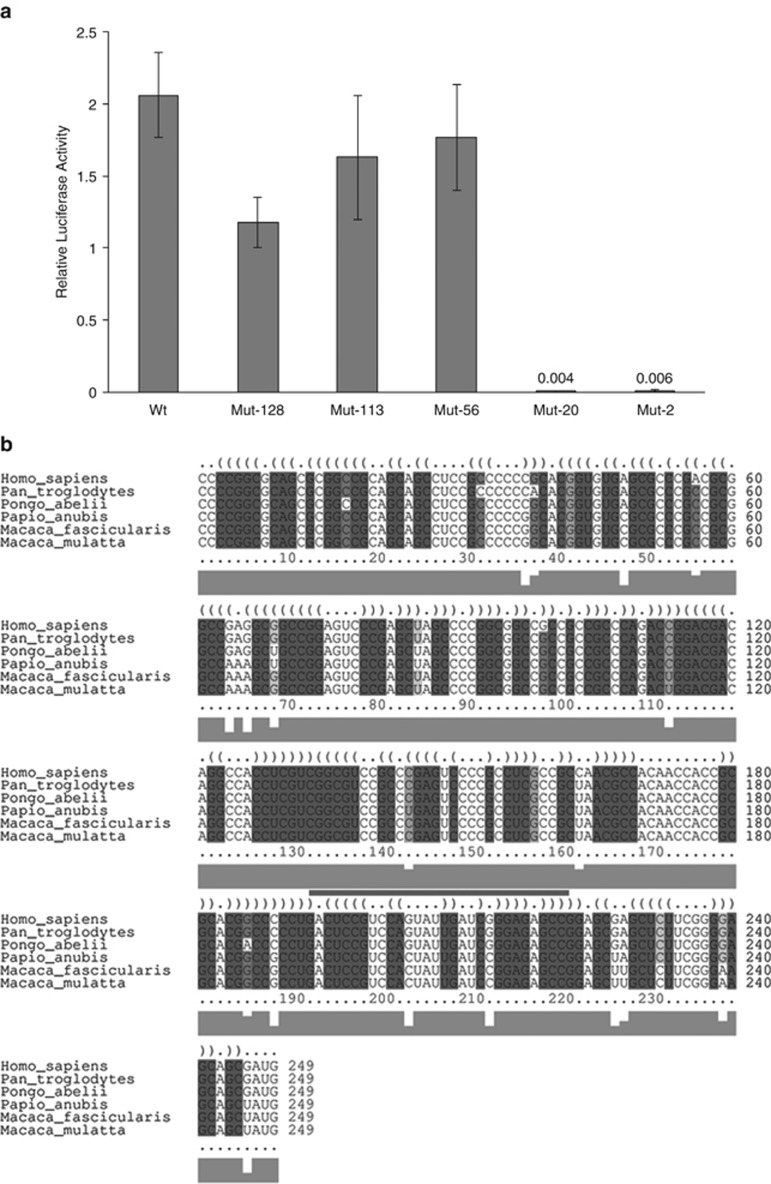
The ribosome enters the EGFR 5′ UTR near the start codon. Five mutant versions of the plasmid pR-EGFR-F were created that introduced AUG start codons at different positions within the EGFR 5′ UTR (mutants are named according to the position of the introduced AUG relative to the wild-type AUG). The introduced AUGs are out-of-frame with the downstream luciferase gene, therefore, if the ribosome bound to the sequence upstream of one of these, a non-functional protein would result and quantified luciferase levels would reduce to background. (**a**) A 24-well plate was seeded with SH-SY5Y cells. These were allowed to recover overnight before being transfected with 200 ng/well of the mutant or wild-type reporter plasmids. After 24 h, luciferase levels were assayed. Mean and s.d of at least three replicates are shown. (**b**) Alignment of primate EGFR 5′ UTR refseq sequences using Locarna (Will *et al.*^47, 48^ and Smith *et al.*^46^) suggests that the ribosome entry site (blue bar) may lie within a conserved stem loop structure near the start codon.

By introducing a series of upstream out-of-frame (relative to luciferase) AUG start codons into the EGFR 5′ UTR sequence we have identified that the 40S ribosomal subunit enters the IRES between 23 and 56 nucleotide (nt) upstream of the authentic AUG start codon (Figures 3a and b). Introduction of out-of-frame AUGs further than 56 nt from the luciferase start codon have no effect on luciferase expression, indicating that the 40S ribosomal subunit enters at a position downstream; introduction of an out-of-frame AUG at position −20 completely abolishes luciferase expression, suggesting that the ribosome enters before this position. An alignment of refseq^45^ primate EGFR 5′ UTR sequences using LocARNA^46, 47, 48^ suggests that the ribosome entry region, between positions −56 and −20, may be located within a conserved stem loop close to the start codon (Figure 4b).

These findings suggest an opportunity to exploit the hypoxic control of EGFR translation to develop new targeted therapeutics. Current therapies such as tyrosine kinase inhibitors or antibodies, which indiscriminately target EGFR, are associated with a number of unpleasant and potentially serious side effects.^49^ Since hypoxia in otherwise healthy patients is generally restricted to tumour masses,^50^ targeting only the hypoxic expression of EGFR offers a way of restricting EGFR inhibition to tumour masses, allowing normal expression of EGFR elsewhere in the body and a consequent reduction in systemic side effects. Although hypoxia targeting drug delivery systems have been under development for a number of years,^50, 51^ understanding the mechanisms that allow hypoxic expression of drug targets is particularly important in allowing us to directly target gene expression specifically in cancer cells.
